# A Hybrid Interweave–Underlay Countrywide Millimeter-Wave Spectrum Access and Reuse Technique for CR Indoor Small Cells in 5G/6G Era

**DOI:** 10.3390/s20143979

**Published:** 2020-07-17

**Authors:** Rony Kumer Saha

**Affiliations:** Radio and Spectrum Laboratory, KDDI Research Inc., 2-1-15 Ohara, Fujimino-shi, Saitama 356-8502, Japan; ro-saha@kddi-research.jp

**Keywords:** 28 GHz, 6G, countrywide, millimeter-wave, spectrum access and reuse, hybrid interweave–underlay, cognitive radio, small cell, mobile network, in-building

## Abstract

In this paper, we propose a hybrid interweave–underlay spectrum access and reuse technique for the dynamic spectrum access and reuse of the countrywide 28 GHz millimeter-wave (mmWave) spectrum to in-building small cells of each mobile network operator (MNO) in a country. For the spectrum access, the proposed technique explores both interweave and underlay spectrum access techniques, whereas, for the spectrum reuse, it considers reusing the countrywide spectrum to each three-dimensional (3D) cluster of small cells in a building. To access the countrywide spectrum, each MNO is considered by paying a licensing fee following its number of subscribers. We present the 3D clustering of in-building of small cells and derive average capacity, spectral efficiency (SE), and energy efficiency (EE). We then perform extensive numerical and simulation results and analyses for an MNO of a country consisting of four MNOs. It is shown that, for no spectrum reuse to in-building small cells, the proposed technique improves average capacity and SE by 3.63 and 2.42 times, respectively, whereas EE improves by 72.79%. However, for vertical spatial reuse of six times (as an example) to small cells in a building, average capacity, SE, and EE improve further by 21.77 times, 14.51 times, and 95.66%, respectively. Moreover, the proposed technique can satisfy SE and EE requirements for sixth-generation (6G) mobile systems by horizontal spatial reuse of the countrywide spectrum to small cells of about 40.62%, 9.37%, and 6.25% less buildings than that required by the traditional static licensed spectrum access (SLSA) technique.

## 1. Introduction

### 1.1. Background 

The ever-increasing demands for network capacity and high data rates due to overwhelming rich multimedia services lead to the scarcity of radio frequency spectrum in mobile communications. Moreover, the traditional non-dynamic or static allocation of spectrum, which allocates a certain portion of the spectrum specified for a country to each mobile network operator (MNO), causes inefficient spectrum utilization. Spectrum extension is a direct way to address the scarcity of the spectrum of an MNO. Hence, unlike allocating a portion of the countrywide spectrum by using the traditional static spectrum allocation, the spectrum of an MNO can be increased by allocating the whole countrywide spectrum. In this regard, due to the availability of a large amount of spectrum bandwidth, the millimeter-wave (mmWave) spectrum is considered as an effective candidate to address the scarcity of spectrum of existing MNOs. However, accessing the same countrywide spectrum by all MNOs in a country, at a time, can generate co-channel interference (CCI), which can be managed by accessing the countrywide spectrum dynamically by each MNO. 

In line with the abovementioned, cognitive radio (CR) is a key enabling technology where dynamic spectrum access is a major function [1]. In CR, interweave and underlay are two main dynamic spectrum access strategies [2]. The major difference between these two strategies relates to how primary users (PUs) and secondary users (SUs) interact [3]. More specifically, in interweave spectrum access, SUs can access the unused spectrum of PUs opportunistically without generating any interference to PUs. This necessitates SUs to monitor periodically the spectrum of PUs to detect the idle spectrum to communicate, which may reduce the effectiveness of spectrum utilization. However, the interweave spectrum access takes advantages of SUs for not being suffered by interference from the primary transmission, as well as for transmitting at the maximum power without incurring any limit (typically bounded by an interference power constraint set by PUs), resulting in achieving superior system performance such as outage probability [4]. 

On the other hand, in the underlay spectrum access, an SU can share the spectrum of PUs simultaneously subject to limiting its transmission power to an interference power constraint set by PUs. Limiting the transmission power of an SU leads to a reduction in the channel capacity and radio coverage of the secondary network. However, due to utilizing the spectrum at any time, the effectiveness of spectrum utilization is improved [4]. Hence, considering the benefits of both interweave and underlay spectrum access techniques, the combination of these two spectrum access techniques can maximize spectral efficiency (SE) and energy efficiency (EE) [5]. Due to this, hybrid cognitive radio networks (CRNs), particularly hybrid interweave–underlay spectrum access, have been proposed as a means to improve the performance of SUs. 

### 1.2. Related Study 

In the existing literature, the hybrid interweave–underlay spectrum access in CRNs has been proposed under different contexts. For example, the authors in Reference [6] have proposed a hybrid underlay–interweave mode enabled CRN scheme. In Reference [7], the authors have proposed a hybrid interweave–underlay spectrum access scheme using spectrum sensing in the 5 GHz license-exempt spectrum. Further, the authors in Reference [8] have introduced a novel hybrid CR system that combines the conventional interweave and underlay paradigms to enhance the chances of the SUs to access the spectrum. Furthermore, in Reference [9], the authors have applied the joint spectrum access and power control in a hybrid interweave–underlay CR system and proposed a modified spectrum access formulation based on the probabilistic spectrum access scheme, to reduce the impact of threshold estimation uncertainty on the performance of the hybrid CR system. Moreover, in Reference [10], the authors have presented a hybrid interweave/underlay CR system by jointly considering spectrum sensing and data transmission.

The performance analysis of the hybrid underlay–interweave access has also been addressed by several studies. For instance, the authors in Reference [11] have provided expressions that allow the performance comparison of the interweave and underlay modes under a unified network setup. Moreover, in Reference [12], the authors have presented the downlink capacity region of a secondary network in a multiuser spectrum sharing system for a hybrid underlay–interweave paradigm. Furthermore, in Reference [2], the authors have examined the throughput performance of a hybrid Multiple-Input-Multiple-Output (MIMO) interweave/underlay CR system on an analytical basis. Whereas, the authors in Reference [13] have proposed two algorithms to optimize power allocation among SUs, successful in accessing the spectrum by using a hybrid interweave/underlay access scheme to maximize SE while respecting the power budget constraints.

Besides, the hybrid underlay–interweave spectrum access has been studied for numerous issues in the existing literature. Accordingly, the authors in Reference [14] have studied mutual interference in the context of hybrid interweave–underlay cognitive communication. In Reference [4], the authors have studied a hybrid interweave–underlay spectrum access system that integrates amplify-and-forward relaying. Moreover, the authors in Reference [3] have addressed a hybrid setup for CR based on the Gaussian interference channel where the secondary user can use both interweave and underlay strategies.

Furthermore, the hybrid underlay–interweave access has also been found to gain attention by researchers, in the context of cooperative communications. In line with this, the authors in Reference [15] have proposed a spectrum efficient CRN that employs a hybrid underlay–interweave mode of CRNs for SUs under cooperative communication. In Reference [16], the authors have proposed two optimal power allocation strategies for hybrid interweave–underlay cognitive cooperative radio networks, to maximize channel capacity and minimize outage probability. Further, in Reference [17], the authors have proposed power allocation strategies to maximize the sum-rate and minimize the outage probability of a hybrid two-way cognitive cooperative radio network with hybrid interweave–underlay spectrum access in the presence of imperfect spectrum sensing.

### 1.3. Contribution

However, mobile data are generated mostly indoors, particularly in dense urban areas, including a large number of multistory buildings installed with small cells. Hence, large spectrum availability, as well as its utilization, is critical to serving high data rates and capacity in such buildings. In this regard, due to the availability of a large amount of spectrum and a suitable signal propagation characteristic indoors, reusing the same countrywide high-frequency mmWave spectrum spatially more than once to small cells within a multistory building is considered to be a promising candidate to increase the available spectrum, as well as to ensure efficient utilization of the available spectrum, to serve high data rates and capacity indoors. In line with this, unlike existing works, a hybrid interweave–underlay spectrum access and reuse technique for sharing and reusing the countrywide mmWave spectrum with in-building small cells of all MNOs in a country to increase the available spectrum and its utilization within multistory buildings can play a vital role in serving high capacity and data rates indoors. We address this issue in this paper. 

In addressing the abovementioned, in this paper, we propose a hybrid interweave–underlay spectrum access and reuse technique for the dynamic spectrum access and reuse of the countrywide 28 GHz mmWave spectrum to in-building small cells of each MNO in a country. For the spectrum access, the proposed technique takes advantage of exploring both interweave spectrum access (i.e., no CCI from PUs, as well as small cells transmitting at the maximum power) and underlay spectrum access (i.e., utilizing the spectrum by SUs at any time) techniques. More specifically, using the interweave access, a small cell of a serving-MNO located in an apartment of a building can access opportunistically the whole 28 GHz spectrum specified for a country by operating at the maximum transmission power so long as no user equipment (UE) of any interfering-MNO in the country is present at the same time in the same apartment of the small cell. However, if a UE of any interfering-MNO is present in the same apartment of the small cell, following the underlay access, the corresponding small cell of the serving-MNO reduces its transmission power and operates simultaneously at the same spectrum with the small cell of the corresponding interfering-MNO in the same apartment. However, for the spectrum reuse, it takes advantage of reusing the whole countrywide 28 GHz mmWave spectrum to small cells of an MNO in a building more than once. In doing so, three-dimensional (3D) clusters of small cells in a building are formed, and each consists of a set of small cells subject to satisfying CCI constraints both in the intra-floor and inter-floor levels. 

### 1.4. Organization 

We organize the paper as follows. In Section 2, we present the system architecture and the propose our hybrid interweave–underlay spectrum access and reuse technique. In Section 3, we first present the 3D cluster formation of in-building small cells in the 28 GHz mmWave spectrum. We then present the analytical model for the proposed technique and derive average capacity, SE, and EE performance metrics for an MNO by employing the proposed technique, as well as the traditional static licensed spectrum access (SLSA) technique. In Section 4, we present default simulation parameters and assumptions and perform extensive numerical and simulation results and analyses for an MNO of a country (consisting of four MNOs) to show outperformance of the proposed technique over the traditional SLSA technique by varying both the vertical spatial reuse and horizontal spatial reuse factors. Finally, we demonstrate that the proposed technique can satisfy both SE and EE requirements for sixth-generation (6G) mobile systems. We conclude the paper in Section 5.

## 2. System Architecture and the Proposed Technique

### 2.1. System Architecture 

**Figure 1:**
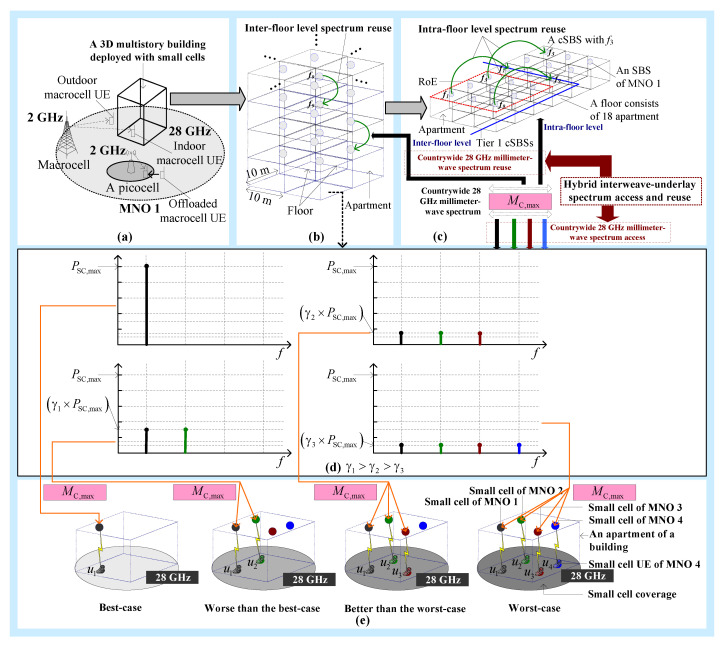
A system architecture consisting of four mobile network operators (MNOs), including MNO 1, MNO 2, MNO 3, and MNO 4, in a country: (**a**) detailed architecture of MNO 1; (**b**) inter-floor level spectrum reuse for MNO 1; (**c**) intra-floor level spectrum reuse for MNO 1; (**d**) variation in the transmission power of small-cell base stations (SBSs) within an apartment in accordance with the existence of their user equipments (UEs).; (**e**) illustration of an example countrywide 28 GHz mmWave spectrum sharing with an in-building SBS of MNO 1. $u_{1}$, $u_{2}$, $u_{3}$, and $u_{4}$ denote a UE of MNO 1, MNO 2, MNO 3, and MNO 4, respectively. $M_{C,\max}$ denotes countrywide 28 GHz mmWave spectrum. $P_{\mathrm{SC},\max}$ denotes the maximum transmission power of an in-building SBS. $\gamma_{1}$, $\gamma_{2}$, and $\gamma_{3}$ denote scaling factors for the transmission power of an in-building SBS such that $\gamma_{1}>\gamma_{2}>\gamma_{3}$; $\left\{f_{1},f_{2},f_{3},\ldots ,f_{9}\right\}$ denotes a set of orthogonal frequencies in the countrywide 28 GHz mmWave spectrum.

Figure 1 shows a system architecture where we consider that four MNOs (i.e., MNO 1, MNO 2, MNO 3, and MNO 4) are operating in a country, even though the actual number of MNOs may vary. Assume that each MNO has a similar system architecture consisting of three types of base stations (BSs), namely macrocell BSs (MBSs), picocell BSs (PBSs), and small-cell BSs (SBSs). For simplicity, we show the detailed architecture of only one MNO (i.e., MNO 1) in Figure 1a. All SBSs are deployed only within 3D multistory buildings, with each building serving one UE at a time. Both in-building SBSs and PBSs are located within the coverage of an MBS. All macrocell UEs of an MBS are served either by the MBS itself or by any PBSs within its coverage. SBSs within each building are considered operating at the 28 GHz mmWave spectrum, whereas MBSs and PBSs are operating at the 2 GHz spectrum, as shown in Figure 1a. 

Like the authors of Reference [18], we assume that each multistory building consists of a set of square-grid apartments of size 10 × 10 m^2^ per floor, and there are multiple floors per building, as shown in Figure 1b. For simplicity, we assume that each building has the same number of small cells, though, in practice, the number of apartments per building varies from one to another. Further, we assume that a small cell of an MNO serves only one UE at any time and is located on the ceiling of an apartment. Given the size of an apartment, we assume that each MNO can have a maximum of one SBS. Further, we assume that each MNO has an SBS in each apartment of each multistory building, though, in practice, the existence of an SBS of any MNO is random. We consider minimum CCI constraints at a co-channel SBS (cSBS) in both the intra-floor and the inter-floor levels to define a 3D cluster of SBSs such that the same countrywide 28 GHz mmWave spectrum can be reused to SBSs in both the inter-floor level (Figure 1b) and the intra-floor level (Figure 1c). Subject to satisfying the CCI constraints and considering only the first-tier of cSBSs, the same spectrum cannot be reused to a set of SBSs in the intra-floor and inter-floor levels, which defines the region of exclusion (RoE), as shown in Figure 1c, for the intra-floor level only. The set of SBSs in RoE constitutes a 3D cluster such that the same countrywide 28 GHz mmWave spectrum can be reused for each 3D cluster of SBSs in a building. 

Furthermore, we assume that each MNO can access the whole countrywide 28 GHz mmWave spectrum, which can be reused further to its SBSs per 3D cluster in each building, following a hybrid interweave–underlay spectrum access and reuse technique presented in the following section. Figure 1d,e shows an example for sharing the countrywide 28 GHz mmWave spectrum with an in-building SBS of MNO 1 by varying its transmission power in accordance with the existence of UEs of other MNOs to limit the aggregate CCI at any UE in an apartment to a predefined CCI threshold set by MNOs to operate under the underlay spectrum access, using the proposed technique. Hence, in Figure 1e, the best case refers to a scenario of no CCI to UE *u*_1_ of MNO 1 when no UEs of other MNOs are present in the same apartment as that of *u*_1_ such that the SBS of MNO 1 serving *u*_1_ can transmit at the maximum power of $P_{\mathrm{SC},\max}$ under the interweave spectrum access. Likewise, the worst-case scenario for the maximum CCI occurs when all three UEs of other MNOs are present in the apartment with *u*_1_, resulting in reducing the transmission power of the SBS of MNO 1 under the underlay spectrum access to the lowest level of $\left(\gamma_{3}\times P_{\mathrm{SC},\max}\right)$, where $\gamma_{1}>\gamma_{2}>\gamma_{3}$. We describe in detail the transmission power control mechanism for an SBS in the following section.

### 2.2. Proposed Technique

We propose a hybrid interweave–underlay spectrum access and reuse (HIUSAR) technique for the dynamic spectrum access and reuse of the countrywide 28 GHz mmWave spectrum to in-building small cells of each MNO in a country stated as follows. The licensed 28 GHz mmWave spectrum specified for a country can be allowed to share with small cells one per apartment in a building of any MNO (termed as serving-MNO) subject to operating each small cell of the (s-MNO) at the maximum transmission power if no UE of any other MNOs (termed as interfering-MNOs) is present (i.e., using the interweave spectrum access technique), whereas at a reduced transmission power if a UE of any interfering-MNO (i-MNO) is present (i.e., using the underlay spectrum access technique), within the same apartment of the corresponding small cell of s-MNO. Additionally, by forming 3D clusters of small cells of the s-MNO in a building, the whole countrywide mmWave spectrum of all MNOs can be allowed to reuse each 3D cluster. The reduced transmission power of small cells for sharing and the 3D clustering of small cells for reusing the countrywide mmWave spectrum are varied in accordance with the predefined interference thresholds set by the i-MNOs and the s-MNO, respectively. Moreover, each MNO pays for accessing the countrywide spectrum licensing fee in accordance with its number of subscribers updated at a certain time interval to ensure fairness in paying the countrywide spectrum licensing fee by each MNO.

Hence, for spectrum sharing, the proposed technique takes advantage of exploring both the interweave and underlay spectrum access techniques. Using the interweave access, a small cell of an s-MNO located in an apartment of a building can access opportunistically the whole 28 GHz spectrum specified for a country by operating at the maximum transmission power, so long as no UE of any i-MNO in the country is present at the same time, in the same apartment of the small cell. However, if a UE of any i-MNO is present in the same apartment of the small cell, following the underlay access, the corresponding small cell of the s-MNO reduces its transmission power immediately, making it compliant with a predefined interference threshold varied in accordance with the presence of UEs of different i-MNOs in the same apartment of the small cell. In addition, the proposed technique takes advantage of reusing the whole countrywide 28 GHz mmWave spectrum to small cells of the s-MNO in a building more than once. In doing so, 3D clusters of small cells in a building are formed, and each consists of a set of small cells subject to satisfying CCI constraints both in the intra-floor and inter-floor levels. The whole countrywide spectrum is then allocated to each 3D cluster of small cells, to reuse it more than once in a building. 

Hence, the proposed technique shares the countrywide 28 GHz spectrum to small cells in each building of an MNO by exploring hybrid interweave and underlay cognitive radio spectrum access techniques to extend the available mmWave spectrum of the MNO. In addition, by exploiting the countrywide 28 GHz mmWave spectrum shared with small cells in a building of an MNO, the same countrywide spectrum is reused more than once per building by forming 3D clusters of small cells of the MNO. In this regard, the small cells of an s-MNO keep sensing to detect the presence of UEs for each i-MNO, to update the corresponding spectrum access mode of operation to either the interweave access or the underlay access (compliant with the required change in the transmission power of them) such that CCI constraint to a UE of the respective i-MNO can be guaranteed. More specifically, each small cell of an s-MNO needs to switch only between two modes of its transmission power, either the maximum or one of the reduced values. For switching, both reactive and proactive spectrum sensing approaches can be applied to detect the usage of the countrywide spectrum. In the reactive approach, an s-MNO performs spectrum sensing mechanisms to detect the usage on the countrywide spectrum, whereas in the proactive approach, based on the knowledge of the traffic model of UEs of an i-MNO, the arrival of UEs can be predicted [19] to reduce the transmission power of the small cell of s-MNO beforehand. Moreover, since the spectrum sensing and switching of the transmission power of small cells of all MNOs in an apartment of a building are performed by small cells locally, in a distributed manner, no additional control signaling overhead is generated in the network of any MNO, due to applying the proposed technique. 

Unlike the traditional SLSA technique where a portion of the countrywide spectrum is allocated to an MNO exclusively, in the proposed technique, each MNO can get access opportunistically to the whole countrywide spectrum. This results in overcoming the lack of a sufficient amount of spectrum of an MNO to serve its user demand due to accessing a large amount of countrywide spectrum by sharing with other MNOs in a country. Further, it addresses the issue of the under-utilized or unused spectrum of an MNO from allowing other MNOs to share with the countrywide spectrum such that the overall countrywide spectrum utilization is improved. Moreover, since each MNO can pay the spectrum licensing fee in accordance with its number of subscribers, an MNO with a smaller number of subscribers can pay less, while an MNO with a greater number of subscribers needs to pay more. Such a fair spectrum licensing fee for each MNO results in reducing the overall cost per unit achievable capacity (i.e., bps) countrywide. The proposed technique described above applies to all MNOs in a country.

## 3. Problem Formulation 

### 3.1. Three-Dimensional Cluster Formation of In-Building Small Cells in 28 GHz Millimeter-Wave 

We adopt the interference modeling for in-building small cells operating at the 28 GHz mmWave proposed in Reference [18] and describe it in brief in the following. The omnidirectional multi-frequency combined polarization *close-in free space reference distance with frequency-dependent path loss exponent* (CIF) large-scale path-loss at a distance *d* from a mmWave signal transmitter is given by Reference [20], as follows:

(1) $$PL[\mathrm{dB}]=61.38+17.97\log_{10}\left(d\right)+X_{\Delta }$$

where $X_{\Delta }$ is the Gaussian random variable with standard deviation, Δ, which represents the large-scale signal variation about the mean path loss due to the shadowing effect. Let $d_{\mathrm{CCI},\mathrm{intra}}$ denote the distance between a small cell UE (sUE) and a cSBS in the intra-floor level. Then, using the above path loss model in (1), the normalized intra-floor interference from a cSBS to an sUE is given as follows:

(2) αCCI,intranor(dCCI,intra) =(dmindCCI,intra)1.797

where $d_{\min}$ denotes the minimum distance between an sUE and the nearest cSBS.

Similarly, if $d_{\mathrm{CCI},\mathrm{inter}}$ denotes the distance between an sUE and a cSBS in the inter-floor level, the normalized inter-floor interference from a cSBS located on a floor other than the floor of an sUE itself is given by the following:

(3) αCCI,internor(dCCI,inter) =10−0.1 αfloor(dCCI,inter)×(dmindCCI,inter)1.797

where $\alpha_{\mathrm{floor}}\left(d_{\mathrm{CCI},\mathrm{inter}}\right)$ denotes the penetration loss of any floor. Following Reference [21], for a typical reinforced concrete floor, we assume that the penetration loss is 55 dB for the first floor as a worst-case analysis [22,23].

Let $\alpha_{\mathrm{CCI},\mathrm{intra}}^{\mathrm{nor},\mathrm{total}}$ and $\alpha_{\mathrm{CCI},\mathrm{inter}}^{\mathrm{nor},\mathrm{total}}$ denote, respectively, the normalized total interference effect in the intra-floor level and the inter-floor level. Then, the normalized total CCI at an sUE due to cSBSs of both the intra-floor and inter-floor levels is given by the following:

(4) $$\alpha_{\mathrm{CCI}}^{\mathrm{nor},\mathrm{total}}=\alpha_{\mathrm{CCI},\mathrm{intra}}^{\mathrm{nor},\mathrm{total}}+\alpha_{\mathrm{CCI},\mathrm{inter}}^{\mathrm{nor},\mathrm{total}}$$

Now, if $I_{\mathrm{optimal},\mathrm{intra}}$ and $I_{\mathrm{optimal},\mathrm{inter}}$ denote, respectively, the optimal value of CCI in the intra-floor level and the inter-floor level, the total optimal value of CCI set by the MNO is given by the following:

(5) $$I_{\mathrm{optimal},\mathrm{total}}=I_{\mathrm{optimal},\mathrm{intra}}+I_{\mathrm{optimal},\mathrm{inter}}$$

Hence, by using (4) and (5), we can obtain the optimal size of a 3D cluster of SBSs when satisfying the following condition.

(6) $$\alpha_{\mathrm{CCI}}^{\mathrm{nor},\mathrm{total}}\leq I_{\mathrm{optimal},\mathrm{total}}$$

However, finding the optimal size of a 3D cluster of SBSs $\Theta_{3D}{}^{*}$ corresponds to finding the optimal minimum distance between cSBSs of $d_{\mathrm{CCI},\mathrm{intra}}{}^{*}$ in the intra-floor level and $d_{\mathrm{CCI},\mathrm{inter}}{}^{*}$ in the inter-floor level.

Let $I_{\max,\mathrm{intra}}$ and $I_{\max,\mathrm{inter}}$ denote, respectively, the maximum number of cSBSs for an sUE in the intra-floor level and the inter-floor level. Then, $d_{\mathrm{CCI},\mathrm{intra}}{}^{*}$ and $d_{\mathrm{CCI},\mathrm{inter}}{}^{*}$ can be expressed as follows.

(7) dCCI,intra*=dmin×(Imax,intraIoptimal,intra)1.797−1

(8) dCCI,inter*≥dmin×(10−0.1 αfloor(dCCI,inter)×(Imax,interIoptimal,inter))1.797−1

where $I_{\max,\mathrm{intra}}=8$ for the first-tier of intra-floor level cSBSs. Similarly, for the first-tier of inter-floor level cSBSs, $I_{\max,\mathrm{inter}}=2$ for the double-sided cSBSs (one on top, whereas the other on a bottom floor from that of an sUE), and $I_{\max,\mathrm{inter}}=1$ for the single-sided cSBSs (either one on top, or a bottom floor from that of an sUE). 

Let $d_{\mathrm{floor}}$ denotes the height of a floor, and *a* denotes the side length of a square apartment. Let $\Theta_{\mathrm{intra}}$ and $\Theta_{\mathrm{inter}}$ denote, respectively, the maximum number of SBSs in the region of exclusion corresponding to satisfying $d_{\mathrm{CCI},\mathrm{intra}}{}^{*}$ and $d_{\mathrm{CCI},\mathrm{inter}}{}^{*}$, which can be can be expressed as follows.

(9) $$\Theta_{\mathrm{intra}}={\left(\mathrm{ceil}\;\left(d_{\mathrm{CCI},\mathrm{intra}}^{*}+\left(a/2\right)/a\right)\right)}^{2}$$

(10) $$\Theta_{i\mathrm{nter}}=\mathrm{ceil}\;\left(d_{\mathrm{CCI},\mathrm{inter}}^{*}/d_{\mathrm{floor}}\right)$$

Hence, the optimal size of a 3D cluster of small cells, $\Theta_{3D}{}^{*}$, and the corresponding spectrum reuse factor per multistory building,$\varepsilon_{\mathrm{RF}}$, are given, respectively, as follows.

(11) $$\Theta_{3D}{}^{*}=\left(\Theta_{i\mathrm{ntra}}\times \Theta_{i\mathrm{nter}}\right)$$

(12) $$\varepsilon_{\mathrm{RF}}=S_{F}/\Theta_{3D}{}^{*}$$

where *S*_F_ denotes the number of small cells in a building. The detailed modeling of $\Theta_{3D}{}^{*}$ in (11) can be found in Reference [18]. 

### 3.2. Mathematical Analysis

#### 3.2.1. Proposed Technique 

Assume that *O* denotes the maximum number of MNOs of a country such that o∈O={1,2,…,O}. Let $M_{C,\max}$ denote countrywide 28 GHz mmWave spectrum defined in terms of the number of resource blocks (RBs) where an RB is equal to 180 kHz. Assume that *L* denotes the number of buildings per macrocell such that l∈{1,2,…,L}. Recall that *S*_F_ denotes the number of small cells per 3D building such that $s\in \left\{1,2,\ldots ,S_{F}\right\}$, where *S*_F_ is assumed the same for all buildings. Let *S*_M_ denote the number of macrocells, and *S*_P_ denotes the number of picocells per macrocell of each MNO. Moreover, let *T* denote simulation run time with the maximum time of *Q* (in time step each lasting 1 ms) such that *T* = {1, 2, 3, …, *Q*}. Denote $P_{\mathrm{MC}}$ and $P_{\mathrm{PC}}$, respectively, as the transmission power of a macrocell and a picocell. 

Assume that each MNO *o* has an SBS in each apartment, and each SBS can serve the maximum of one UE at a time. Assume that there are four MNOs in a country such that o∈O={1,2,3,4}. Thus, the maximum of four different UEs, each from one MNO, may exist at once in an apartment. Let $P_{\mathrm{SC},\mathrm{int},o}$ and $P_{\mathrm{SC},\mathrm{und},o}$ denote, respectively, the transmission power of an SBS of MNO *o* when operating under the interweave and underlay spectrum access techniques. Let $P_{\mathrm{SC},\max,o}$ and $P_{\mathrm{SC},\mathrm{red},o}$ denote, respectively, the maximum transmission power and the reduced transmission power of an SBS of MNO *o* when operating under the interweave and underlay spectrum access techniques such that $P_{\mathrm{SC},\max,o}>P_{\mathrm{SC},\mathrm{red},o}$. Assume that $\forall o\;P_{\mathrm{SC},\max,o}=P_{\mathrm{SC},\max}$ and $\forall o\;P_{\mathrm{SC},\mathrm{red},o}=P_{\mathrm{SC},\mathrm{red}}$. 

Let $I_{\mathrm{thr},\mathrm{und},o}$ denote the predefined value of the maximum aggregate CCI that can be caused by an SBS of an MNO *o* to UEs of other MNOs ***O***\*o* when operating under the underlay spectrum access technique. Assume that $\forall o\;I_{\mathrm{thr},\mathrm{und},o}=I_{\mathrm{thr},\mathrm{und}}$. Since the value of $I_{\mathrm{thr},\mathrm{und},o}$ varies in accordance with the available number of UEs of MNOs ***O***\*o* within the coverage of an SBS of an MNO *o* in an apartment,$I_{\mathrm{thr},\mathrm{und},o}$ can then be expressed in terms of $P_{\mathrm{SC},\max}$, as follows.

(13) $$I_{\mathrm{thr},\mathrm{und},o}=\left\{\begin{array}{ll} \left(\gamma_{1}\times P_{\mathrm{SC},\max}\right), & \left(\left|O\right|\backslash o\right)=1\\ \left(\gamma_{2}\times P_{\mathrm{SC},\max}\right), & \left(\left|O\right|\backslash o\right)=2\\ \left(\gamma_{3}\times P_{\mathrm{SC},\max}\right), & \left(\left|O\right|\backslash o\right)=3 \end{array}\right\}$$

where $\gamma_{1}>\gamma_{2}>\gamma_{3}$. Note that, when operating under the underlay spectrum access technique, MNOs cause CCI mutually to one another. Hence, assume that $I_{\mathrm{thr},\mathrm{und}}=\left(0.3\times P_{\mathrm{SC},\max}\right)$. Then, in (13), $\gamma_{1}$, $\gamma_{2}$, and $\gamma_{3}$ are given by $\gamma_{1}=0.3$, $\gamma_{2}=0.15$, and $\gamma_{3}=0.10$ such that the total CCI at a UE of any MNO o∈O={1,2,3,4} is limited by $\left(0.3\times P_{\mathrm{SC},\max}\right)$.

However, if κ denotes the interference channel gain, then the transmission power of an SBS of an MNO *o* can be adapted with $I_{\mathrm{thr},\mathrm{und}}$ as follows [24].

(14) $$P_{\mathrm{SC},\mathrm{und},o}=\left\{\begin{array}{ll} P_{\mathrm{SC},\mathrm{red},o}, & \left(\kappa \times P_{\mathrm{SC},\mathrm{red},o}\right)\leq I_{\mathrm{thr},\mathrm{und}}\\ \left(I_{\mathrm{thr},\mathrm{und}}/\kappa \right), & \left(\kappa \times P_{\mathrm{SC},\mathrm{red},o}\right)>I_{\mathrm{thr},\mathrm{und}} \end{array}\right\}$$

Let $u_{1}$, $u_{2}$, $u_{3}$, and $u_{4}$ denote, respectively, a UE of MNO 1, MNO 2, MNO 3, and MNO 4 in an apartment. Each UE has two states for existence (i.e., a UE of an MNO may either exist or not) in an apartment. Let the binary digits 1 and 0 denote, respectively, the existence and nonexistence of a UE of an MNO *o* in an apartment such that four UEs can coexist in an apartment in a maximum of $2^{4}$ possible ways, as shown in Table 1. Assume that the existence of four UEs in an apartment for each possible way as shown in Table 1 is equally likely. Hence, given the existence (i.e., the binary state 1) of a UE of an MNO *o* (i.e., an s-MNO) over the observation time of |T|=Q, UEs of other MNOs ***O****\o* (i.e., i-MNOs) can coexist with the UE of MNO *o* in a maximum of eight possible ways, as shown in the lower half of Table 1; each occurs with a probability of 1/8 and hence persists for Q/8 during the observation time *Q* in an apartment.

For example, for a UE $u_{1}$ of MNO 1 as an s-MNO, the maximum possible combinations that $u_{1}$ can coexist with other UEs $u_{2}$, $u_{3}$, and $u_{4}$ of MNO 2, MNO 3, and MNO 4 as i-MNOs, respectively, are $\left\{u_{1}\right\}$, $\left\{u_{1},u_{2}\right\}$, $\left\{u_{1},u_{3}\right\}$, $\left\{u_{1},u_{4}\right\}$, $\left\{u_{1},u_{2},u_{3}\right\}$, $\left\{u_{1},u_{2},u_{4}\right\}$, $\left\{u_{1},u_{3},u_{4}\right\}$, and $\left\{u_{1},u_{2},u_{3},u_{4}\right\}$, where each occurs with a probability of 1/8 in an apartment. Since an SBS of each MNO is allowed to operate at the whole countrywide mmWave spectrum of $M_{C,\max}$ RBs, using the above possible combinations in Table 1 for the coexistence of $u_{1}$ with other UEs (i.e., $u_{2}$, $u_{3}$, and $u_{4}$) in an apartment, the operating time of the SBS of MNO *o* serving $u_{1}$ corresponds to the variation in the number of UEs of ***O****\o* in an apartment over the observation time of |T|=Q is given by the following:

(15) $$t_{\mathrm{SC},o}=\left\{\begin{array}{ll} Q/8, & \left(\left|O\right|\backslash o\right)=0\\ 3Q/8, & \left(\left|O\right|\backslash o\right)=1\\ 3Q/8, & \left(\left|O\right|\backslash o\right)=2\\ Q/8, & \left(\left|O\right|\backslash o\right)=3 \end{array}\right\}$$

Hence, using (15), we can now define the transmission power of the SBS serving $u_{1}$ of MNO *o* in an apartment as follows.

(16) $$P_{\mathrm{SC},o}=\left\{\begin{array}{ll} P_{\mathrm{SC},\max}, & Q/8\\ \left(\gamma_{1}\times P_{\mathrm{SC},\max}\right), & 3Q/8\\ \left(\gamma_{2}\times P_{\mathrm{SC},\max}\right), & 3Q/8\\ \left(\gamma_{3}\times P_{\mathrm{SC},\max}\right), & Q/8 \end{array}\right\}$$

Hence, from (16), for an equally likely probability of each combination for the existence of $u_{1}$ of MNO *o* = 1 with UEs $u_{2}$, $u_{3}$, and $u_{4}$ of other MNOs O\o in an apartment, the SBS serving $u_{1}$ operates under the interweave spectrum access during Q/8, whereas under the underlay spectrum access during 7Q/8, it operates over the observation time of |T|=Q. 

The received signal-to-interference-plus-noise ratio (SINR) at RB = *i* in transmission time interval (TTI) = *t* at a UE of an MNO *o* can be expressed as follows:

(17) $$\rho_{t,i,o}=\left(P_{t,i,o}/\left(N_{t,i,o}^{s}+I_{t,i,o}\right)\right)\;.\;H_{t,i,o}$$

where $P_{t,i,o}$ is the transmission power, $N_{t,i,o}^{s}$ is the noise power, $I_{t,i,o}$ is the total interference signal power, and $H_{t,i,o}$ is the link loss for a link between a UE and a BS of an MNO *o* at RB = *i* in TTI = *t.* $H_{t,i,o}$ can be expressed in dB as follows:

(18) $$H_{t,i,o}\left(\mathrm{dB}\right)=(G_{t}+G_{r})-(L_{F}+PL_{t,i,o})+(LS_{t,i,o}+SS_{t,i,o})$$

where $\;\left(G_{t}+G_{r}\right)$ and $L_{F}$ are, respectively, the total antenna gain and connector loss.$LS_{t,i,o}$, $SS_{t,i,o}$, and $PL_{t,i,o}$, respectively, denote large-scale shadowing effect, small-scale Rayleigh or Rician fading, and distance-dependent path loss between a BS and a UE of an MNO *o* at RB = *i* in TTI = *t.*

Using Shannon’s capacity formula, a link throughput at RB = *i* in TTI = *t* for an MNO *o* in bps per Hz is given by [25,26] the following:

(19) $$\sigma_{t,i,o}\left(\rho_{t,i,o}\right)=\left\{\begin{array}{ll} 0, & \;\rho_{t,i,o}<-10\;\mathrm{dB}\\ \beta \;\log_{2}\left(1+10^{\left(\rho_{t,i,o}\left(\mathrm{dB}\right)/10\right)}\right)\;, & -10\;\mathrm{dB}\leq \rho_{t,i,o}\leq 22\;\mathrm{dB}\\ 4.4, & \;\rho_{t,i,o}>22\;\mathrm{dB} \end{array}\right\}$$

where *β* denotes the implementation loss factor.

Let $M_{\mathrm{MBS},o}$ denote the 2 GHz spectrum in RBs of a macrocell for an MNO *o*. Then, the total capacity of all macrocell UEs for an MNO *o* can be expressed as follows:

(20) $$\sigma_{\mathrm{MBS},o}=\sum_{t=1}^{Q}\sum_{i=1}^{M_{\mathrm{MBS},o}}\sigma_{t,i,o}\left(\rho_{t,i,o}\right)$$

where σ and ρ are responses over $M_{\mathrm{MBS},o}$ RBs of all macro UEs in *t* ∈ ***T*** for an MNO *o*.

Now, if all SBSs in each building simultaneously serve in *t* ∈ ***T***, using Table 1, the aggregate capacity served by an SBS of an MNO *o* (i.e., an s-MNO) can be found as follows. Using (15) and (16), let $P_{\mathrm{SC},\mathrm{int},\mathrm{RB}}$ denote the transmission power per RB (corresponding to $P_{\mathrm{SC},\max}$) of an SBS of MNO *o* when operating under the interweave spectrum access technique for (|O|\o)=0 in an apartment. Likewise,$P_{{\mathrm{SC},\mathrm{und}}_{\gamma_{1}},\mathrm{RB}}$, $P_{{\mathrm{SC},\mathrm{und}}_{\gamma_{2}},\mathrm{RB}}$, and $P_{{\mathrm{SC},\mathrm{und}}_{\gamma_{3}},\mathrm{RB}}$ denote, respectively, the transmission power per RB (corresponding to $\left(\gamma_{1}\times P_{\mathrm{SC},\max}\right)$, $\left(\gamma_{2}\times P_{\mathrm{SC},\max}\right)$, and $\left(\gamma_{3}\times P_{\mathrm{SC},\max}\right)$, respectively) of an SBS of an MNO *o* when operating under the underlay spectrum access technique for (|O|\o)=1, (|O|\o)=2, and (|O|\o)=3, respectively, in an apartment.

Assume that $\rho_{t,i,o,\mathrm{int}}$ denote the received SINR at RB = *i* in TTI = *t* at a UE of an SBS of MNO *o* when operating at the power $P_{t,i,o}$ of $P_{\mathrm{SC},\mathrm{int},\mathrm{RB}}$ under the interweave access technique. Similarly, let $\rho_{t,i,o,{\mathrm{und}}_{\gamma_{1}}}$, $\rho_{t,i,o,{\mathrm{und}}_{\gamma_{2}}}$, and $\rho_{t,i,o,{\mathrm{und}}_{\gamma_{3}}}$ denote, respectively, the received SINR at RB = *i* in TTI = *t* at a UE of an SBS of MNO *o* when operating at the power $P_{t,i,o}$ of $P_{{\mathrm{SC},\mathrm{und}}_{\gamma_{1}},\mathrm{RB}}$, $P_{{\mathrm{SC},\mathrm{und}}_{\gamma_{2}},\mathrm{RB}}$, and $P_{{\mathrm{SC},\mathrm{und}}_{\gamma_{3}},\mathrm{RB}}$, respectively, under the underlay access technique. Let $\sigma_{t,i,o,\mathrm{int}}$, $\sigma_{t,i,o,{\mathrm{und}}_{\gamma_{1}}}$, $\sigma_{t,i,o,{\mathrm{und}}_{\gamma_{2}}}$, and $\sigma_{t,i,o,{\mathrm{und}}_{\gamma_{3}}}$ denote, respectively, the link throughput corresponding to $\rho_{t,i,o,\mathrm{int}}$, $\rho_{t,i,o,{\mathrm{und}}_{\gamma_{1}}}$, $\rho_{t,i,o,{\mathrm{und}}_{\gamma_{2}}}$, and $\rho_{t,i,o,{\mathrm{und}}_{\gamma_{3}}}$. 

Using (17) and (19), the capacity served by an SBS of MNO *o* using the interweave access at the countrywide spectrum of $M_{C,\max}$ in *t* ∈ ***T*** is given by the following:

(21) $$\sigma_{s,o,\mathrm{int}}=\sum_{t=1}^{\left(Q/8\right)}\sum_{i=1}^{M_{C,\max}}\sigma_{t,i,o,\mathrm{int}}\left(\rho_{t,i,o,\mathrm{int}}\right)$$

Moreover, the capacity served by an SBS of MNO *o* using the underlay access at the countrywide spectrum of $M_{C,\max}$ in *t* ∈ ***T*** is given by the following:

(22) $$\sigma_{s,o,\mathrm{und}}=\left(\begin{array}{l} \sum_{t=1}^{\left(3Q/8\right)}\sum_{i=1}^{M_{C,\max}}\sigma_{t,i,o,{\mathrm{und}}_{\gamma_{1}}}\left(\rho_{t,i,o,{\mathrm{und}}_{\gamma_{1}}}\right)\\ +\sum_{t=1}^{\left(3Q/8\right)}\sum_{i=1}^{M_{C,\max}}\sigma_{t,i,o,{\mathrm{und}}_{\gamma_{2}}}\left(\rho_{t,i,o,{\mathrm{und}}_{\gamma_{2}}}\right)\\ +\sum_{t=1}^{\left(Q/8\right)}\sum_{i=1}^{M_{C,\max}}\sigma_{t,i,o,{\mathrm{und}}_{\gamma_{3}}}\left(\rho_{t,i,o,{\mathrm{und}}_{\gamma_{3}}}\right) \end{array}\right)$$

Hence, the overall aggregate capacity served by an SBS of MNO *o* using the proposed hybrid interweave–underlay technique at the countrywide spectrum of $M_{C,\max}$ in *t* ∈ ***T*** is given by the following:

(23) $$\sigma_{s,o,\mathrm{prop}}=\sigma_{s,o,\mathrm{int}}+\sigma_{s,o,\mathrm{und}}$$

Let $S_{F,\mathrm{cl}}$ denote the number of small cells per 3D cluster. Recall that the whole countrywide 28 GHz mmWave spectrum can be reused to each 3D cluster of small cells in a building. Then, the aggregate capacity served by all SBSs per 3D cluster in a building is given by the following:

(24) $$\sigma_{S_{F,\mathrm{cl}},o,\mathrm{prop}}=\sum_{s=1}^{S_{F,\mathrm{cl}}}\sigma_{s,o,\mathrm{prop}}$$

Now, the aggregate capacity served by all SBSs *S*_F_ of MNO *o* in *a building* using the proposed hybrid interweave–underlay technique in *t* ∈ ***T*** is given by the following:

(25) $$\sigma_{S_{F},o,\mathrm{prop}}=\varepsilon_{\mathrm{RF}}\times \sum_{s=1}^{S_{F,\mathrm{cl}}}\sigma_{s,o,\mathrm{prop}}$$

Recall that an MNO *o* pays for the spectrum licensing fee in accordance with the number of its subscribers,$N_{o}$, such that $\sum_{o=1}^{O}N_{o}=N_{C,\max}$, where $N_{C,\max}$ denotes the total number of subscribers countrywide. Let $M_{o}$ denote the amount of 28 GHz mmWave spectrum in RBs of an MNO *o* corresponding to its subscribers, $N_{o}$, such that $\sum_{o=1}^{O}M_{o}=M_{C,\max}$. Further, due to a short distance between a small cell UE and its SBS and a low transmission power of an SBS, we assume similar indoor signal propagation characteristics for all *L* buildings per macrocell for MNO *o*. Then, by linear approximation, the system-level average capacity, SE, and EE for MNO *o* are given for *L* > 1, respectively, as follows.

(26) $$\sigma_{\mathrm{cap},o,\mathrm{prop}}^{\mathrm{sys}}\left(L\right)=\sigma_{\mathrm{MBS},o}+\left(L\times \sigma_{S_{F},o,\mathrm{prop}}\right)$$

(27) $$\sigma_{\mathrm{SE},o,\mathrm{prop}}^{\mathrm{sys}}\left(L\right)=\sigma_{\mathrm{cap},o,\mathrm{prop}}^{\mathrm{sys}}\left(L\right)/\left(\left(M_{\mathrm{MBS},o}+M_{o}\right)\times Q\right)$$

(28) $$\sigma_{\mathrm{EE},o,\mathrm{prop}}^{\mathrm{sys}}\left(L\right)=\frac{\left(\begin{array}{l} \left(\left(S_{P}\times P_{\mathrm{PC}}\right)+\left(S_{M}\times P_{\mathrm{MC}}\right)\right)+\\ \left(L\times \left(\varepsilon_{\mathrm{RF}}\times S_{F,\mathrm{cl}}\times \left(\begin{array}{l} \left(P_{\mathrm{SC},\max}/8\right)+\left(3\times \left(\gamma_{1}\times P_{\mathrm{SC},\max}\right)/8\right)+\\ \left(3\times \left(\gamma_{2}\times P_{\mathrm{SC},\max}\right)/8\right)+\left(\left(\gamma_{3}\times P_{\mathrm{SC},\max}\right)/8\right) \end{array}\right)\right)\right) \end{array}\right)}{\left(\sigma_{\mathrm{cap},o,\mathrm{prop}}^{\mathrm{sys}}\left(L\right)/Q\right)}$$

#### 3.2.2. SLSA technique

In the traditional SLSA technique, each MNO is licensed exclusively for an equal amount of 28 GHz mmWave spectrum of *M* RBs, i.e., $\forall o\;M_{o}=M:\sum_{o=1}^{O}M_{o}=M_{C,\max}$. Then, for SLSA, the system-level average capacity, SE, and EE for MNO *o* for *L*>1 are given, respectively, by the following:

(29) $$\sigma_{\mathrm{cap},o,\mathrm{SLSA}}^{\mathrm{sys}}\left(L\right)=\sigma_{\mathrm{MBS},o}+\left(L\times \sigma_{S_{F},o,\mathrm{SLSA}}\right)$$

where $\sigma_{S_{F},o,\mathrm{SLSA}}=\sum_{s=1}^{S_{F}}\sum_{t\in T}\sum_{i=1}^{M}\sigma_{s,t,i,o}\left(\rho_{s,t,i,o}\right)$

(30) $$\sigma_{\mathrm{SE},o,\mathrm{SLSA}}^{\mathrm{sys}}\left(L\right)=\sigma_{\mathrm{cap},o,\mathrm{SLSA}}^{\mathrm{sys}}\left(L\right)/\left(\left(M_{\mathrm{MBS},o}+M\right)\times Q\right)$$

(31) $$\sigma_{\mathrm{EE},o,\mathrm{SLSA}}^{\mathrm{sys}}\left(L\right)=\left(\begin{array}{l} \left(L\times S_{F}\times P_{\mathrm{SC},\max}\right)\\ +\left(S_{P}\times P_{\mathrm{PC}}\right)+\left(S_{M}\times P_{\mathrm{MC}}\right) \end{array}\right)/\left(\sigma_{\mathrm{cap},o,\mathrm{SLSA}}^{\mathrm{sys}}\left(L\right)/Q\right)$$

Hence, the improvement factors for average capacity, SE, and EE performances due to applying the proposed technique for MNO *o* (i.e., an s-MNO) with respect to that due to applying the SLSA technique can be expressed, respectively, as follows.

(32) $$\sigma_{\mathrm{cap},o,\mathrm{IF}}^{\mathrm{sys}}\left(L\right)=\sigma_{\mathrm{cap},o,\mathrm{prop}}^{\mathrm{sys}}\left(L\right)/\sigma_{\mathrm{cap},o,\mathrm{SLSA}}^{\mathrm{sys}}\left(L\right)$$

(33) $$\sigma_{\mathrm{SE},o,\mathrm{IF}}^{\mathrm{sys}}\left(L\right)=\sigma_{\mathrm{SE},o,\mathrm{prop}}^{\mathrm{sys}}\left(L\right)/\sigma_{\mathrm{SE},o,\mathrm{SLSA}}^{\mathrm{sys}}\left(L\right)$$

(34) $$\sigma_{\mathrm{EE},o,\mathrm{IF}}^{\mathrm{sys}}\left(L\right)=\sigma_{\mathrm{EE},o,\mathrm{prop}}^{\mathrm{sys}}\left(L\right)/\sigma_{\mathrm{EE},o,\mathrm{SLSA}}^{\mathrm{sys}}\left(L\right)$$

## 4. Performance Evaluation 

### 4.1. Default Parameters and Assumptions 

Table 2 shows the default simulation parameters and assumptions used for evaluating the performance of the proposed technique for MNO 1, as an s-MNO. Default simulation assumptions, parameters, and models used for the performance evaluation are in line with the recommendations from the standardization bodies such as the 3rd generation partnership project (3GPP) and International Telecommunication Union-Radiocommunication Sector (ITU-R). For the interweave spectrum access, an SBS is assumed to transmit at its maximum power, whereas, for the underlay spectrum access, we assume that the reduced transmission power of an SBS is upper limited by 30%, 15%, and 10% of its maximum transmission power when the total number of UEs of i-MNOs exists within an apartment is one, two, and three, respectively, i.e., one UE from each i-MNO. Note that these values of the reduced transmission power of an SBS for the underlay spectrum access can be chosen differently other than those stated above based on an operator’s quality-of-service requirements.

Due to emphasizing more on the spectrum management, as well as the number of MNOs countrywide, than other issues such as the density of users and user traffic characteristics, we assume that each small cell of an MNO can serve the maximum of one UE at a time for the simplicity in analysis and closed-form solution. However, the proposed technique applies as well to the scenario of serving multiple users simultaneously by each small cell per MNO, which is out of the scope of this paper. Because of the favorable signal propagation characteristics in indoors and the availability of substantial spectrum bandwidth, the 28 GHz mmWave spectrum is considered as an effective spectrum band for the 5G and the future 6G systems [27]. Likewise, to provide a large coverage and smaller number of handoffs, 2 GHz microwave spectrum is considered for the outdoor microcells and picocells.

Further, due to the less multipath fading effect of high-frequency signals in indoor environments, we consider the line-of-sight (LOS) large-scale path loss model for the 28 GHz mmWave signals within buildings. Similarly, because of a small coverage and less multipath fading effect of an indoor small cell operating at the 28 GHz band, we assume a similar mmWave signal propagation characteristic within each adjacent building. Moreover, though, in practice, the available bandwidth in the 28 GHz band can be as large as more than 1.6 GHz [28], for simplicity, we consider only countrywide 200 MHz spectrum bandwidth for evaluating the performance of the proposed technique. Furthermore, we consider that four MNOs are operating in a country as an example scenario to evaluate the performance of the proposed technique. However, in practice, the number of MNOs in a country can be arbitrary, and the proposed technique applies to any number of MNOs in a country.

Furthermore, we consider a performance evaluation scenario where the existence of any combination of four UEs one from each MNO in an apartment of a building is equally likely for a worse case analysis. Due to considering an equally likely existence of any combination of UEs, an SBS of an MNO can operate at the maximum transmission power under the interweave access during only one, whereas at a reduced transmission power under the underlay access during the rest seven, of the eight possible combinations (Table 1), which result in achieving a worse capacity performance for an observation time *Q*. However, given the randomness in the existence of any combination of four UEs in an apartment, in practice, an SBS of an MNO may operate randomly either at the maximum transmission power for *Q* under the interweave access (for the best case analysis), or at the minimum transmission power for *Q* under the underlay access (for the worst-case analysis), or at any combination of the values of the transmission power of the SBS for *Q*. 

In addition, because of a high external wall penetration loss of a building and a low transmission power of any in-building small cell, the 28 GHz mmWave spectrum can be reused to small cells in adjacent buildings. Moreover, the full buffer model is considered for simplicity, such that resource schedulers can be assumed to have user traffic to serve at any time over the observation period *Q*. Further, because of providing balance performances between throughputs and fairness in radio resource allocations, the proportional fair (PF) resource scheduler is considered. Finally, performance results are generated simulating all assumptions, parameters, and models given in Table 2 by a simulator built by using the computational tool MATLAB R2012b version running on a personal computer. 

### 4.2. Performance Results

**Figure 2:**
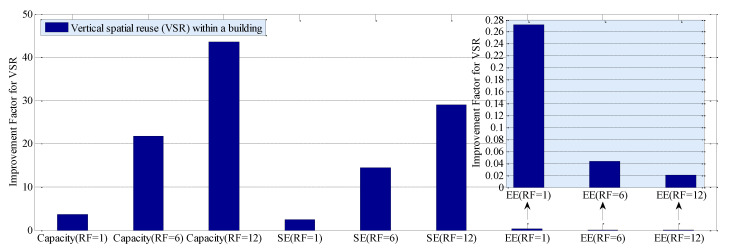
Performance improvement of the proposed technique over the traditional static licensed spectrum access (SLSA) technique due to the variation in spatial reuse factor (RF) vertically within a building, in terms of average capacity, spectral efficiency (SE), and energy efficiency (EE).

Figure 2 shows average capacity, SE, and EE outperformance of the proposed technique over the traditional SLSA technique due to the variation in the vertical spatial reuse (VSR), i.e., reuse factor (RF), of the countrywide spectrum to SBSs of MNO 1 in a building. It can be found from Figure 2 that, in general, average capacity and SE increase linearly, whereas EE improves negative exponentially with an increase in RF. Since the capacity is directly proportional to the available spectrum, by reusing the same spectrum spatially, the capacity can be increased, which in turn increases the SE. As EE is inversely related to the achievable capacity, an increase in capacity results in decreasing the energy required to transfer per unit bit. 

Note that, when considering no reuse of the countrywide spectrum (i.e., RF = 1), the proposed technique improves average capacity and SE by 3.63 and 2.42 times, whereas it reduces the energy required per bit transmission (i.e., EE) by 72.79%. With an increase in the VSR, average capacity, SE, and EE improve further, for example, to 21.77 times for average capacity and 14.51 times for SE, and 95.66% for EE when reusing the countrywide spectrum six times (i.e., RF = 6) to small cells in a building. Hence, VSR can play a significant role in enhancing average capacity, SE, and EE of 5G and beyond mobile systems.

Moreover, the improvement in SE is less than that of average capacity, even though the SE is directly proportional to the capacity. This is because SE, in addition to the capacity, varies inversely with the available spectrum. Since, for the worse-case analysis, we assume that MNO 1 has 40% of the total countrywide subscribers in the proposed technique, MNO 1 needs to pay for 40% of the total countrywide spectrum, in contrast to 25% countrywide spectrum when employing the traditional SLSA technique. Due to an increase in the available spectrum, SE increases less than that of capacity, even though it can access the whole countrywide spectrum. However, as we reduce the spectrum to 25% for paying the licensing fee, SE can vary in proportion with the average capacity in Figure 2.

**Figure 3:**
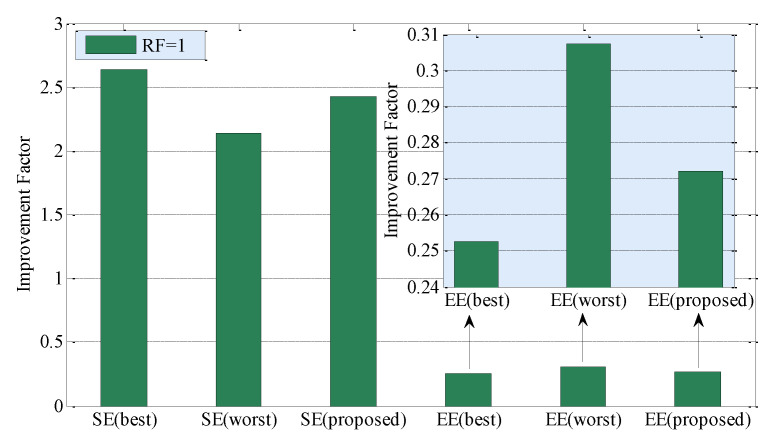
Performance of the proposed technique over the traditional SLSA technique due to the variation in co-channel interference (CCI) experienced by each SBS of MNO 1 located in an apartment of a building for RF = 1 in terms of SE and EE. The best case refers to no CCI, and the worst-case refers to the maximum CCI.

Figure 3 shows SE and EE performances for the best case and the worst case CCI scenarios to evaluate the performance of the proposed technique. From Figure 3, with respect to the traditional SLSA, SE can be improved by 2.64 times in the best case, 2.14 times in the worst case, and 2.42 times by using the proposed technique. Likewise, EE can be improved by 74.76% in the best case, 69.27% in the worst case, and 72.79% by using the proposed technique. Since both SE and EE performance improvement of the proposed technique lies in between that in case of the best-case and worst-case CCI scenarios, the proposed technique can capture the CCI scenario reasonably. Moreover, it justifies as well the accuracy of modeling with an equally likely assumption for the existence of UEs of SBSs of different MNOs in an apartment of a building and the corresponding mutual CCI generated due to operating at the same countrywide spectrum.

**Figure 4:**
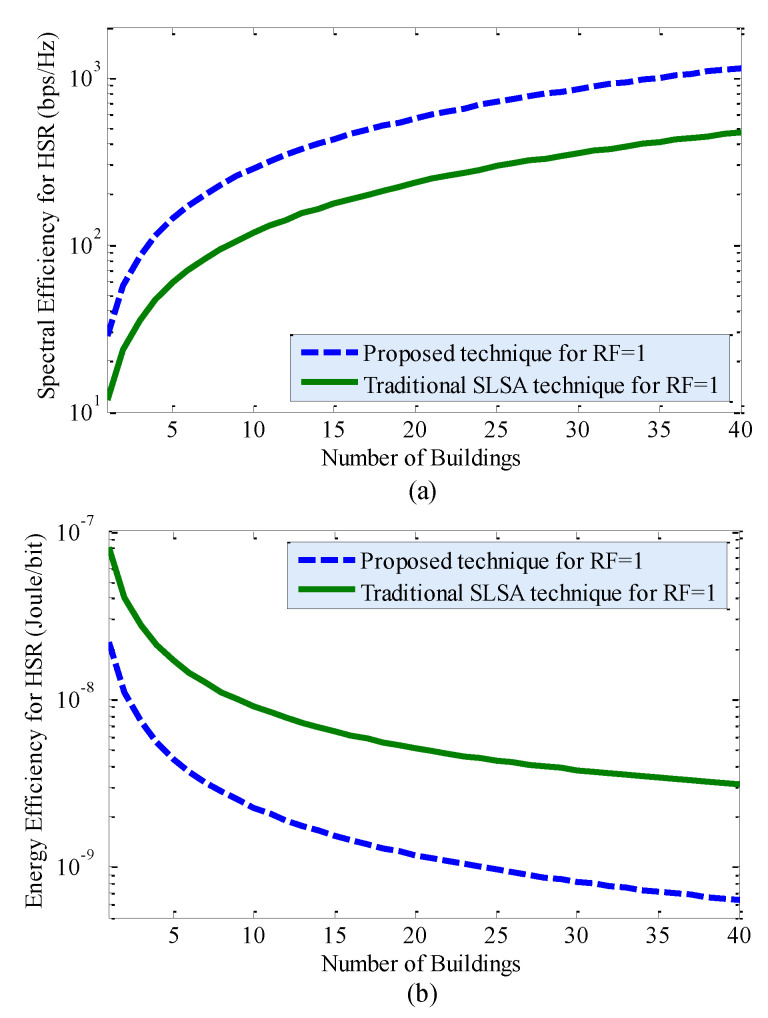
Performance of the proposed technique and the traditional SLSA technique due to the variation in horizontal spatial reuse (HSR) between adjacent buildings of small cells in terms of SE and EE for RF = 1: (**a**) SE performance and (**b**) EE performance.

**Figure 5:**
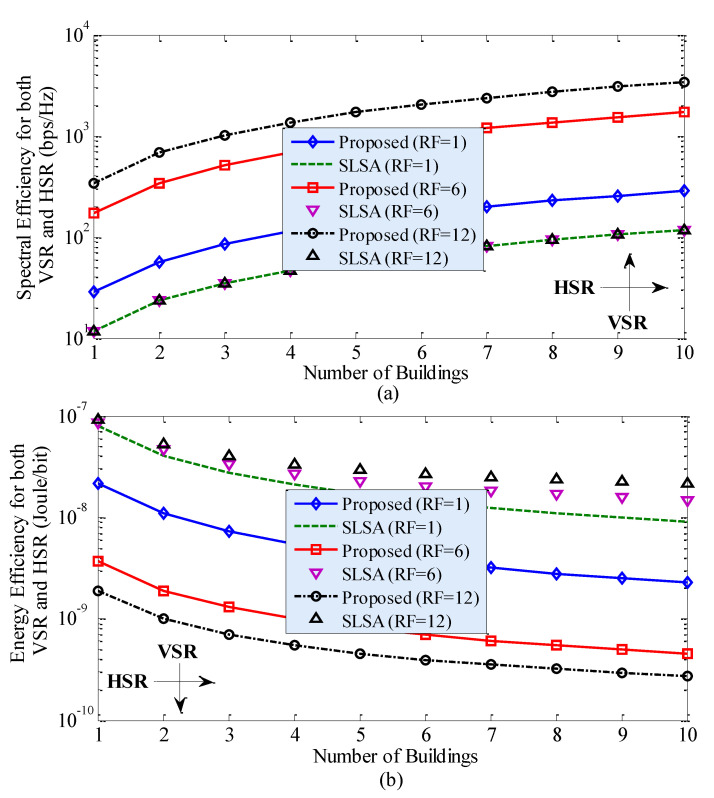
Performance of the proposed technique and the traditional SLSA technique due to the variation in both vertical spatial reuse (VSR) within a building, as well as HSR between adjacent buildings, of small cells in terms of (**a**) SE and (**b**) EE.

Figure 4 shows the SE and EE performances of the proposed technique and the traditional SLSA technique due to the variation in the horizontal spatial reuse (HSR) of the countrywide mmWave spectrum for RF = 1. Note that HSR defines reusing the same countrywide spectrum to multiple building of small cells (i.e., inter-building spectrum reuse), unlike VSR, where the spectrum is reused to small cells by forming 3D clusters within the same building (i.e., intra-building spectrum reuse). It can be found from Figure 4 that, for both techniques, with an increase in HSR (i.e., *L*), SE increases linearly, whereas EE increases rapidly for low values of *L* and gets almost fixed for high values of *L*. However, the proposed technique outperforms the traditional SLSA technique considerably in terms of both SE and EE performances. In short, HSR plays a significant role in enhancing SE as well as EE performances of 5G and beyond systems. Since both VSR in Figure 2 and HSR in Figure 4 can enhance SE and EE performances when applying each of them alone, a joint application of both VSR and HSR of the countrywide spectrum to in-building SBSs can improve SE and EE further by a factor corresponding to the multiplication of RF and *L*, as shown in Figure 5. Figure 5 implies that the massive SE and EE enhancement can be obtained by reusing the countrywide 28 GHz mmWave spectrum to small cells in both intra-building and inter-building scenarios.

### 4.3. Performance Comparison

In line with Reference [33], the future 6G mobile systems are expected to require 10 times average SE (i.e., 270–370 bps/Hz), as well as 10 times average EE [27] (i.e., 0.3 × 10^−6^ Joules/bit), of 5G mobile systems [34,35]. Let $\sigma_{\mathrm{SE}}^{6G}$ = 370 bps/Hz and $\sigma_{\mathrm{EE}}^{6G}$ = 0.3 µJ/b denote, respectively, the required average SE and average EE for 6G mobile systems. Now, using Figure 5, Table 3 shows the required HSR of the countrywide 28 GHz mmWave spectrum in term of the number of buildings of small cells *L* with a variation in the VSR of the countrywide 28 GHz mmWave spectrum per building of small cells in terms of RF when applying the proposed technique, as well as the traditional SLSA technique, to in-building small cells of MNO 1. From Table 3, it can be found that, though the proposed, as well as the traditional SLSA technique, can satisfy SE and EE requirements for 6G mobile systems, the proposed technique requires HSR of the countrywide 28 GHz mmWave spectrum to small cells of 40.62%, 9.37%, and 6.25% less buildings corresponding to VSR of the same spectrum of RF equals 1, 6, and 12, respectively, than that required by the traditional SLSA technique.

## 5. Conclusions

In this paper, we have proposed a hybrid interweave–underlay spectrum access and reuse technique for the dynamic spectrum access and reuse of the countrywide 28 GHz millimeter-wave (mmWave) spectrum to in-building small cells of each MNO in a country. The proposed technique considers sharing the 28 GHz mmWave spectrum specified for a country with small cells, one per apartment, in a building of any MNO subject to operating each of its small cells at the maximum transmission power, using interweave access if no UE is present, whereas at a reduced transmission power, using underlay access, if a UE of any other MNOs is present within the apartment of the corresponding small cell. Moreover, by forming 3D clusters of small cells of the MNO in a building, the whole countrywide mmWave spectrum can be allowed to reuse to each cluster. The reduced transmission power of small cells for sharing and the 3D clustering of small cells for reusing the countrywide mmWave spectrum are varied in accordance with the predefined interference thresholds. Moreover, each MNO pays a licensing fee for accessing the countrywide mmWave spectrum in accordance with its number of subscribers updated at a certain time interval to ensure fairness in paying the countrywide spectrum licensing fee by each MNO. 

We have presented forming 3D clusters of in-building small cells in the 28 GHz mmWave spectrum, as well as the analytical model for the proposed technique. Performance metrics, including average capacity, SE, and EE for an MNO were derived by employing the proposed technique, as well as the traditional static licensed spectrum access (SLSA) technique. Extensive numerical and simulation results and analyses for an MNO of a country with four MNOs were carried out to show the performance improvement of the proposed technique over the traditional SLSA technique by varying both the vertical spatial reuse and horizontal spatial reuse factors. It has been found that, when considering no reuse of the countrywide spectrum, the proposed technique improves average capacity and SE by 3.63 and 2.42 times, respectively, whereas it reduces the energy required per bit transmission (i.e., EE) by 72.79%. Moreover, with an increase in the VSR, average capacity, SE, and EE improve further, for example, 21.77 times for average capacity, 14.51 times for SE, and 95.66% for EE, when reusing the countrywide 28 GHz mmWave spectrum six times to small cells in a building. 

Likewise, with an increase in the HSR, SE increases linearly, whereas EE improves rapidly for low values of *L* and gets almost fixed for high values of *L*. However, the proposed technique outperforms the traditional SLSA technique considerably in terms of both SE and EE performances. Since both VSR and HSR can enhance SE and EE performances when applying each alone, a joint application of both VSR and HSR of the countrywide mmWave spectrum to in-building SBSs can improve SE and EE further by a factor corresponding to the multiplication of the spectrum reuse factor within a building and the number of multistory buildings of small cells. Finally, we have demonstrated that the proposed technique requires HSR of the countrywide 28 GHz mmWave spectrum to small cells of 40.62%, 9.37%, and 6.25% less buildings (corresponding to the reuse factors for the VSR of 1, 6, and 12, respectively) than that required by the traditional SLSA technique to satisfy SE and EE requirements for 6G mobile systems.

## Figures and Tables

**Table 1:** Co-existence and probability distribution for the UE $u_{1}$ of MNO 1 with UEs $u_{2}$, $u_{3}$, and $u_{4}$ of other MNOs O\o in an apartment.

State (*j*)	$u_{1}$	$u_{2}$	$u_{3}$	$u_{4}$	Prob (j)	Prob (O\o)				Spectrum Access
						**0**	**1**	**2**	**3**	
0	0	0	0	0	Not applicable due to the nonexistence of $u_{1}$					
1	0	0	0	1						
2	0	0	1	0						
3	0	0	1	1						
4	0	1	0	0						
5	0	1	0	1						
6	0	1	1	0						
7	0	1	1	1						
8	1	0	0	0	$\frac{1}{8}$	$\frac{1}{8}$	$\frac{3}{8}$	$\frac{3}{8}$	$\frac{1}{8}$	Interweave
9	1	0	0	1						Underlay
10	1	0	1	0						
11	1	0	1	1						
12	1	1	0	0						
13	1	1	0	1						
14	1	1	1	0						
15	1	1	1	1						

**Table 2:** Default parameters and assumptions.

Parameters and Assumptions			Value
*Countrywide perspective*			
**Countrywide 28 GHz spectrum bandwidth**			200 MHz
Countrywide total number of MNOs and subscribers			4 and $N_{C,\max}$
2 GHz spectrum per MNO			10 MHz
Number of subscribers for MNOs 1, 2, 3, and 4 respectively			40%, 30%, 20%, and 10% of $N_{C,\max}$
*For each MNO*			
E-UTRA simulation case^1^			3GPP case 3
Cellular layout^2^, inter-site distance (ISD)^1,2^, transmission direction			Hexagonal grid, dense urban, 3 sectors, per macrocell site, 1732 m, downlink
Carrier frequency^2,5^			2 GHz non-LOS (NLOS) for macrocells and picocells, 28 GHz LOS for all small cells
Number of cells			1 macrocell, 2 picocells, 180 small cells per building
Reduced transmission power factors of an SBS for the underlay spectrum access ($\gamma_{1}$, $\gamma_{2}$, and $\gamma_{3}$ respectively)			30%, 15%, and 10% (of the maximum transmission power of 19 dBm)
Total BS transmission power^1^ (dBm)		46 for macrocell^1,4^, 37 for picocells^1^,	
		19 (when operating under interweave access) for small cells^1,3,4^,	
		13.771, 10.76, and 7.943 (when operating under underlay access) for small cells	
Co-channel small-scale fading model^1,3,5^			Frequency selective Rayleigh for 2 GHz NLOS, none for 28 GHz LOS
Path loss	MBS and a UE^1^	Outdoor macrocell UE	*PL*(dB) = 15.3 + 37.6 log_10_*R*, *R* is in m
		Indoor macrocell UE	*PL*(dB) = 15.3 + 37.6 log_10_*R* + *L*_ow_, *R* is in m and *L*_ow_ = 20 dB
	PBS and a UE^1^		*PL*(dB) = 140.7+36.7 log_10_*R*, *R* is in km
	SBS and a UE^1,2,5^		*PL*(dB) = 61.38+17.97 log_10_*R*, *R* is in m
Lognormal shadowing standard deviation (dB)			8 for MBS^2^, 10 for PBS^1^, and 9.9 for SBS^2,5^
Antenna configuration			Single-input single-output for all BSs and UEs
Antenna pattern (horizontal)			Directional (120^0^) for MBS^1^, omnidirectional for PBS^1^ and SBS^1^
Antenna gain plus connector loss (dBi)			14 for MBS^2^, 5 for PBS^1^, 5 for SBS^1,3^
UE antenna gain^2,3^; Indoor macrocell UE^1^			0 dBi (for 2 GHz), 5 dBi (for 28 GHz, Biconical horn); 35%
UE noise figure^2,3^ and UE speed^1^			9 dB (for 2 GHz) and 10 dB (for 28 GHz), 3 km/hr
Picocell coverage, the total number of macrocell UEs, and macrocell UEs offloaded to all picocells^1^			40 m (radius), 30, 2/15
3D multistory building and SBS models (square- grid apartments):		Number of buildings	*L*
		Number of floors per building	10
		Number of apartments per floor	18
		Number of SBSs per apartment	1
		Area of an apartment	10 × 10 m^2^
Scheduler and traffic model^2^			Proportional Fair and full buffer
Type of SBSs			Closed Subscriber Group femtocell BSs
TTI^1^ and scheduler time constant (*t*_c_)			1 ms and 100 ms
Total simulation run time			8 ms

Taken ^1^ from Reference [29], ^2^ from Reference [30], ^3^ from Reference [31], ^4^ from Reference [32], ^5^ from Reference [20].

**Table 3:** Required HSR of the countrywide 28 GHz mmWave spectrum in *L* with a variation in VSR of the spectrum per building of small cells of MNO 1 in RF to satisfy average SE and EE requirements for 6G mobile systems.

VSR (RF)	HSR (*L*) to Satisfy Both Average SE and EE Requirements for 6G Mobile Systems						
	$\sigma_{\mathrm{SE},o=1}^{\mathrm{sys}}\;\geq \sigma_{\mathrm{SE}}^{6G}$		$\sigma_{\mathrm{EE},o=1}^{\mathrm{sys}}\;\leq \sigma_{\mathrm{EE}}^{6G}$		$\max\left(\sigma_{\mathrm{SE},o=1}^{\mathrm{sys}},\sigma_{\mathrm{EE},o=1}^{\mathrm{sys}}\right)$		
	Proposed	SLSA	Proposed	SLSA	Proposed	SLSA	$\left(\frac{\mathrm{Proposed}}{\mathrm{SLSA}}\right)\%$
1	13	32	1	1	13	32	40.62
6	3				3		9.37
12	2				2		6.25
